# Comparative analysis of chloroplast genomes on Meliaceae species: insights into the evolution and species identification

**DOI:** 10.3389/fpls.2025.1536313

**Published:** 2025-03-11

**Authors:** Zixuan Nie, Jikai Ma, Chengkun Wang, Ming Tang, Ting Jia, Guoxiang Liao, Lu Zhang

**Affiliations:** ^1^ Jiangxi Provincial Key Laboratory of Subtropical Forest Resources Cultivation, College of Forestry, Jiangxi Agricultural University, Nanchang, China; ^2^ 2011 Collaboration Innovation Center of Jiangxi Typical Trees Cultivation and Utilization, College of Forestry, Jiangxi Agricultural University, Nanchang, China; ^3^ Jiangxi Provincial Key Laboratory of Improved Variety Breeding and Efficient Utilization of Native Tree Species, College of Forestry, Jiangxi Agricultural University, Nanchang, China

**Keywords:** Meliaceae, *Toona*, chloroplast genome, comparative analyses, divergence times

## Abstract

**Introduction:**

Meliaceae, a significant group in Sapindales, possesses material and medicinal value due to its applications in timber and bioactive compounds. However, the high morphological diversity of the Meliaceae species and the lack of comparative studies of chloroplast (cp) genomes have led to great challenges in the classification and identification of Meliaceae species.

**Methods:**

In this study, we sequenced the complete cp genomes of three *Toona* species (*Toona fargesii*, *Toona ciliata*, and *Toona sinensis*), and conducted comparative analyses of these cp genomes along with 29 previously published cp genomes of Meliaceae. Additionally, we performed the phylogenetic analyses and estimated the divergence times of Meliaceae.

**Results:**

The cp genomes of 32 Meliaceae species ranged from 158,558 bp to 160,978 bp in length. Specifically, the cp genomes of *Toona* varied from 159,242 bp to 159,617 bp in length. These cp genomes contained a total of 135 unique genes, comprising 90 protein-coding, 8 rRNA, and 37 tRNA genes. Divergence time estimation revealed that the Meliaceae family diverged into two subfamilies (Cedreloideae and Melioideae) approximately 72.92 Ma (95% HPD: 60.62-87.01 Ma) in the Late Cretaceous. The diversification of Cedreloideae (47.86 Ma, 95% HPD: 47.42-48.22 Ma) occurred later than that of Melioideae (66.60 Ma, 95% HPD: 55.41-79.73 Ma). Furthermore, comparative genomic analysis identified 52 to 116 simple sequence repeats (SSRs) and twelve highly variable regions (HVRs) found among the cp genomes of Meliaceae.

**Discussion:**

Divergence time estimation indicates that most Meliaceae species have a relatively recent origin, with rapid divergence occurring during the Late Oligocene or Early Miocene epochs. Comparative analysis of cp genomes revealed that Meliaceae exhibits relative conservation in terms of cp genome size, inverted-repeat (IR) boundary, genome structure, HVRs and codon patterns. Although differences exist between the Cedreloideae and Melioideae subfamilies, the overall similarity remains notably high. Furthermore, the *ycf*1, *trn*K-*rps*16, and *ndh*F-*rpl*32 regions exhibited the highest nucleotide polymorphism within Meliaceae, while the *rpl*22 gene displayed significant genetic diversity within both subfamilies. As candidate molecular markers, these regions may effectively distinguish among species. These findings not only provide insights into the evolution and species identification but also establish a scientific foundation for future systematics within Meliaceae.

## 1 Introduction

Meliaceae is a family of flowering plants that includes both trees and shrubs. It comprises 51 genera and 778 species and is widely distributed across tropical and subtropical regions (Laino Gama et al., 2021). Moreover, the majority of Meliaceae members are commercially valued timber trees, primarily belonging to the subfamily Cedreloideae (such as *Khaya, Swietenia*, *Entandrophragma, Cedrela*, and *Toona*) (Koenen, 2011). Furthermore, the Meliaceae family encompasses several economically valuable species with edible, medicinal, and ornamental values (such as *Azadirachta indica, Lansium domesticum*, and *Melia azedarach*). The diversity of fruit, floral, and seed morphologies within Meliaceae is remarkable and frequently influenced by environmental factors. The leaves include two morphologies: pinnately compound and simple (rarely); the inflorescences are typically thyrses, while racemose and spicate forms are relatively rare; the anthers are generally fused to form a tubular structure; the fruits of this family encompass capsules, berries, drupes, and nuts (rarely) (Yadav et al., 2015). Consequently, the phylogeny and infrageneric division of Meliaceae have been contentious, attracting extensive research. Originally, Pennington and Styles (1975) proposed that the Meliaceae family be divided into four subfamilies (Melioideae, Cedreloideae, Quivisianthoideae, and Capuronianthoideae), based on morphological characteristics. This concept was refuted by Muellner et al. (2003), who proposed to retain only two subfamilies (Cedreloideae and Melioideae). They suggested that the Capuronianthus subfamily be classified within the Cedreloideae, and the Quivisianthe subfamily be classified within the Melioideae (Muellner and Mabberley, 2008). The majority of available genetic and morphological evidence supports the classification of Meliaceae into two subfamilies. In a recent study, the classification perspective of Oyedeji Amusa et al. (2024) based on molecular systematics and anatomical data was consistent with Muellner et al. (2003).

*Toona* (Endl.) M. Roem., a significant lineage belonging to the Cedreloideae subfamily, has free stamens that are adnate to an androgynophore, different from other genera in Meliaceae. It is primarily distributed in Eastern, Southern, and Southeast Asia, as well as Eastern Australia (Mabberley et al., 1995), with approximately 15 species globally. In China, the genus includes four species: *T. sinensis*, *T. ciliata*, *T. fargesii*, and *T. sureni*. This genus has been widely utilized for over 250 years and is considered one of the most significant tropical and subtropical hardwoods (Edmonds, 1993). These trees are characterized by their fast growth, straight and well-proportioned form, and attractive wood texture. The obvious distinction between heartwood and sapwood grants them the admirable title of “Chinese mahogany” (Jia et al., 2024). Among these, *T. sinensis* is recognized as a traditional Chinese woody vegetable, serving as a source of timber, as well as having edible and medicinal functions. It has high economic value and development prospects, leading to its widespread cultivation in China. Additionally, three other *Toona* species are sporadically distributed in China. Meanwhile, their populations have been severely reduced due to habitat fragmentation and shrinkage from over-harvesting, utilization, and exploitation of agriculture and industry. Therefore, these species have been classified as threatened (Fu and Jin, 1992). Enhancing the conservation and sustainable utilization of these species is imperative.

Chloroplasts are important organelles responsible for photosynthesis and several metabolic processes in plants, algae, and certain protists. Most chloroplast (cp) genomes possess a tetrameric structure, including four parts: a large single-copy region (LSC), a small single-copy region (SSC), and two inverted-repeat regions (IRA and IRB) (Liu et al., 2022). Variations in the size and orientation of IRA and IRB primarily result in alterations to cp genome size and the appearance of duplicate genes. The cp genome exhibits greater conservation in terms of genetic structure, number, and composition compared to nuclear and mitochondrial genomes (Petit et al., 2005). Owing to these specific characteristics, the cp genome has been extensively utilized on the species identification, phylogeny, and evolution within Meliaceae (Lu et al., 2022). Previous studies employing phylogenetic methods have utilized partial sequences, variable regions, or multi-locus sequences. Muellner et al. (2011) evaluated the *rpo*C1, *rpo*B, and *acc*D regions within the cp genomes of Meliaceae. Unfortunately, these genetic regions were insufficient for accurate species identification. Koenen et al. (2015) constructed a phylogenetic tree for Meliaceae using the cp genome fragments (*rbc*L, *mat*K, *rps*16, and *ycf*1), which exhibited increased support rates and enhanced topological stability compared to earlier studies. Nevertheless, these sequences lack sufficient information for distinguishing among similar species (Daniell et al., 2016). To elucidate evolutionary relationships among closely related species, complete cp genomes have been employed. Lu et al. (2022) indicated that *Toona* and *Cedrela* belong to the subfamily Cedreloideae, with *T. ciliata* and *T. sinensis* displaying a close genetic relationship. Guo et al. (2018) revealed that *Xylocarpus rumphii* is more closely related to *Xylocarpus moluccensis* and *Xylocarpus granatum*. Additionally, comparative analyses of the cp genomes can identify variations to develop molecular markers for species identification, conservation genetics, and phylogenetic analysis. Four highly variable regions (HVRs) have been identified as molecular markers for distinguishing among the five Meliaceae species (Mader et al., 2018). Tan et al. (2023) reported that two genes and four non-coding gene regions exhibited high variability between mangrove and non-mangrove species. However, these studies have primarily concentrated on a limited number of Meliaceae species, leaving the variability in the cp genomes of other species unclear.

Currently, the cp genomes of 34 species across 17 genera within Meliaceae have been published, such as *Entandrophragma cylindricum*, *Cedrela odorata*, *T. sinensis*, *M. azedarach*. Among them, *A. indica* was the first species within Meliaceae to have its cp genome documented in GenBank (Accession Number: KF986530). In this research, we sequenced the cp genomes of three species, including *T. sinensis*, *T. ciliata*, and *T. fargesii.* Furthermore, we re-annotated previously published Meliaceae cp genomes from NCBI. These cp genomes were utilized for phylogenetic analyses, estimation of divergence time, and comparative analysis. Our specific objectives were as follows: (1) to supply newly sequenced complete cp genomes of the *Toona* genus, with the cp genome of *T. fargesii* being reported for the first time; (2) to construct the phylogenetic relationships and infer the divergence times in the evolutionary history of Meliaceae; (3) to compare the sequences and structures of cp genomes within Meliaceae; and (4) to identify the HVRs and SSRs as new barcodes that can distinguish species within Meliaceae.

## 2 Materials and methods

### 2.1 Plant material and DNA isolation

Fresh leaves of *T. fargesii*, *T. ciliata*, and *T. sinensis* utilized in this study were collected from Zhangzhou City, Fujian Province (25°55’N, 116°57’E), Baoshan City, Yunnan Province (25°04’N, 99°06’E), and Nanchang City, Jiangxi Province (28°46’N, 115°49’E), respectively. Voucher specimens were deposited at the College of Forestry, Jiangxi Agricultural University (JXAU), Nanchang, Jiangxi Province. Approximately 5 g of fresh leaves were selected from each material, and total genomic DNA was extracted using an improved CTAB method (Doyle and Doyle, 1987). The purity and concentration of the DNA were assessed using agarose gel electrophoresis and a NanoDrop 2000 microspectrophotometer. After meeting the requirements of sequencing, the DNA was submitted to the Illumina HiSeq 2000 platform at Novogene Bioinformatics Technology Co. Ltd. (Beijing, China), applying the paired-end (PE) 150 bp reads.

### 2.2 Genome assembly and annotation

FastQC v0.19.7 was employed to evaluate the quality of the raw sequencing data. We obtained clean data with a Q20/Q30 score greater than 96/90. With k-mers of 55, 87, and 121, the remaining clean reads were assembled using GetOrganelle v1.7.5 (Jin et al., 2020). Using the published *T. sinensis* cp genome (GenBank accession number: ON244456) as a reference, the cp genomes were annotated using the CPGAVAS2 (Shi et al., 2019) and Geseq (Tillich et al., 2017) platforms. The results were manually checked and adjusted using Geneious v9.0.2 (Kearse et al., 2012). The annotated sequences were uploaded to the NCBI using BankIt to obtain the sequence accession number: PQ634666 (*T. fargesii*), PQ634667 (*T. ciliata*), and PQ634668 (*T. sinensis*). These cp genomes of *Toona* were mapped using the online tool Chloroplot (Zheng et al., 2020).

### 2.3 Structural characterization and comparative chloroplast genome analysis

A total of 32 cp genomes of Meliaceae were obtained from the NCBI database (**Table 1**), among which 29 were published sequences, and 3 were newly sequenced in this study. Geneious v9.0.2 was employed to analyze the cp genomic characteristics of the 32 Meliaceae species, including genome size, length of LSC/SSC/IR, GC content, gene composition, and function. To establish a consensus on the standard and minimize errors, the cp genomes of Meliaceae obtained from NCBI were reannotated.

**Table 1:** Species used in present study.

Number	Species	sp.	Family; Genus	Accession number
1	*Khaya senegalensis*	*K. senegalensis*	Meliaceae; *Khaya*	MZ274125
2	*Khaya grandifoliola*	*K. grandifoliola*	Meliaceae; *Khaya*	MZ274123
3	*Khaya ivorensis*	*K. ivorensis*	Meliaceae; *Khaya*	MZ274124
4	*Khaya anthotheca*	*K. anthotheca*	Meliaceae; *Khaya*	MZ274122
5	*Swietenia mahagoni*	*S. mahagoni*	Meliaceae; *Swietenia*	NC_040009
6	*Swietenia macrophylla*	*S. macrophylla*	Meliaceae; *Swietenia*	MH348156
7	*Xylocarpus rumphii*	*X. rumphii*	Meliaceae; *Xylocarpus*	MH330687
8	*Xylocarpus moluccensis*	*X. moluccensis*	Meliaceae; *Xylocarpus*	MH330688
9	*Xylocarpus granatum*	*X. granatum*	Meliaceae; *Xylocarpus*	MH348155
10	*Carapa guianensis*	*Ca. guianensis*	Meliaceae; *Carapa*	MF401522
11	*Entandrophragma utile*	*E. utile*	Meliaceae; *Entandrophrag*ma	MZ274121
12	*Entandrophragma cylindricum*	*E. cylindricum*	Meliaceae; *Entandrophragma*	MZ274120
13	*Entandrophragma angolense*	*E. angolense*	Meliaceae; *Entandrophragma*	MZ274118
14	*Entandrophragma candollei*	*E. candollei*	Meliaceae; *Entandrophragma*	MZ274119
15	*Lovoa swynnertonii*	*Lo. swynnertonii*	Meliaceae; *Lovoa*	MZ274130
16	*Lovoa trichilioides*	*Lo. trichilioides*	Meliaceae; *Lovoa*	MZ274129
17	*Toona fargesii*	*T. fargesii*	Meliaceae; *Toona*	PQ634666
18	*Toona ciliata*	*T. ciliata*	Meliaceae; *Toona*	PQ634667
19	*Toona sinensis*	*T. sinensis*	Meliaceae; *Toona*	PQ634668
20	*Cedrela odorata*	*Ce. odorata*	Meliaceae; *Cedrela*	MG724915
21	*Leplaea laurentii*	*L. laurentii*	Meliaceae; *Leplaea*	MZ274127
22	*Leplaea thompsonii*	*L. thompsonii*	Meliaceae; *Leplaea*	MZ274128
23	*Leplaea cedrata*	*L. cedrata*	Meliaceae; *Leplaea*	MZ274126
24	*Turraeanthus africanus*	*Tu. africanus*	Meliaceae; *Turraeanthus*	MZ274131
25	*Lansium domesticum*	*La. domesticum*	Meliaceae; *Lansium*	MT937184
26	*Aglaia odorata*	*Ag. odorata*	Meliaceae; *Aglaia*	MN106246
27	*Aphanamixis polystachya*	*Ap. polystachya*	Meliaceae; *Aphanamixis*	MN106249
28	*Cipadessa cinerascens*	*Ci. cinerascens*	Meliaceae; *Cipadessa*	MN126582
29	*Heynea velutina*	*H. velutina*	Meliaceae; *Heynea*	MW246152
30	*Melia azedarach*	*M. azedarach*	Meliaceae; *Melia*	MT460410
31	*Azadirachta siamensis*	*A. siamensis*	Meliaceae; *Azadirachta*	OK037101
32	*Azadirachta indica*	*A. indica*	Meliaceae; *Azadirachta*	OK037102

### 2.4 Phylogenetic analyses

The cp genomes of 32 Meliaceae species were employed to elucidate their phylogenetic relationships, with *Leitneria floridana* (Simaroubaceae, *Leitneria*) serving as the outgroup (GenBank accession number of NC_030482). The cp genomes were aligned by MAFFT v7.520 (Katoh et al., 2002), and the optimal tree-building model was subsequently determined by the ModelFinder function of PhyloSuite v1.2.3 (Zhang et al., 2020). The phylogenetic tree was constructed using the maximum likelihood (ML) approach with 1000 bootstrap replicates in IQ-tree v1.6.8 (Nguyen et al., 2015). This study referred to the classification proposed by Muellner et al. (2003) and Oyedeji Amusa et al. (2024) to avoid confusion.

### 2.5 Divergence time analysis

The approximate divergence times among Meliaceae were estimated using MCMCTree (Puttick, 2019). Four credible fossils that are widely used in molecular dating analysis of Meliaceae (Koenen et al., 2015) were used as calibration points. Calibration point information is as follows: (1) the stem node of Cedreloideae at 47.8 Ma (Chandler, 1964). (2) the stem age of Cedreleae (28.1 Ma) (McGinite, 1953). (3) the stem node of *Swietenia* (22.5 Ma) (Castañeda Posadas and Cevallos Ferriz, 2007). (4) the stem node of the ‘*La. Domesticum-Ap. Polystachya’* clade (23 Ma) (Conran et al., 2014). The uncorrelated lognormal relaxed clock model was selected, and the GTR substitution model was used as the phylogenetic tree model. The convergence of MCMCTree was assessed using Tracer v1.7.2 (Rambaut et al., 2018), and the effective sample size (ESS) value was verified to be above 200. A burn-in of 20% of generations was discarded. The results were visualized by using Figtree v1.4.4 (Rambaut, 2007).

### 2.6 Genome structural variations and sequence divergences

The alterations in the IR boundaries of cp genomes within Meliaceae were analyzed using IRscope (Amiryousefi et al., 2018). The mVISTA online tool (Brudno et al., 2003) employing the Shuffle-LAGAN model was utilized to visualize the variable regions for comparative analysis of Meliaceae cp genomes, with *Khaya senegalensis* (GenBank accession number: MZ274125) serving as the reference. Additionally, cp genome sequences of Meliaceae were aligned using MAFFT v7.520 for further study. The nucleotide polymorphism (Pi) was computed using DnaSP software v6.12.03 (Rozas et al., 2017) with a sliding window length of 600 bp and a step size of 200 bp.

### 2.7 Simple sequence repeats analysis

Simple sequence repeats (SSRs) were analyzed using the MISA online tool (Beier et al., 2017). The minimum threshold of repeats was set to 10 (mononucleotides), 5 (dinucleotides), 4 (trinucleotides), 3 (tetranucleotides), 3 (pentanucleotides), and 3 (hexanucleotides). In addition, the minimum distance between SSRs was set to 100 bp.

### 2.8 Codon usage analysis

To avoid bias, we excluded protein-coding genes (PCGs) shorter than 300 bp and repetitive using Geneious v9.0.2. A total of 52 coding sequences were retained. CodonW v1.4.2 was utilized to calculate the relative synonymous codon usage (RSCU) (Wu et al., 2007) of Meliaceae for an examination of codon usage preference. The results were visualized using the ChiPlot online tool (https://www.chiplot.online/).

## 3 Results

### 3.1 Chloroplast genome structure and features

**Figure 1:**
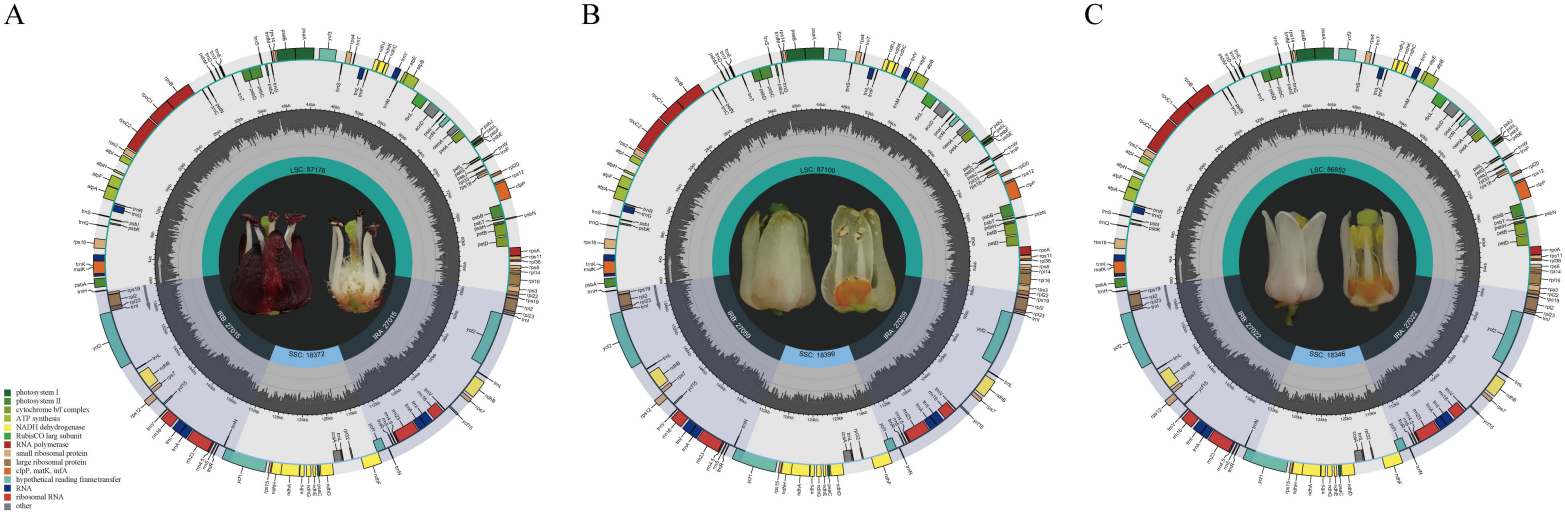
Chloroplast genome maps of **(A)** *T. fargesii*, **(B)** *T. ciliata* and **(C)** *T. sinensis*. The different colored rectangles on the large ring represent different kinds of genes. Genes located outside the large ring are transcribed in a counterclockwise direction, while those inside the large ring are transcribed in a clockwise direction. The dark gray area of the small ring indicates the G and C content.

The cp genome sizes of Meliaceae ranged from 158,558 bp (*Ce. odorata*) to 160,978 bp (*Aglaia odorata*), with the former exhibiting the shortest IR (26,894 bp) and the latter possessing the longest IR (27,089 bp). The cp genome sizes of *Toona* were 159,578 bp, 159,617 bp, and 159,242 bp, respectively, which was within the size observed in the subfamily Cedreloideae (158,558 bp-159,841 bp). All cp genomes of *Toona* exhibit a typical tetrameric structure containing four regions: LSC, SSC, IRB, and IRA (**Figure 1**), consistent with those of Meliaceae. In Meliaceae, the lengths of these regions range from 86,390 bp to 88,197 bp for LSC, 17,967 bp to 18,709 bp for SSC, and 26,894 bp to 27,089 bp for IR, respectively. Furthermore, the cp genome size, LSC length, and SSC length of the subfamily Cedreloideae are shorter than those of the subfamily Melioideae; however, the GC contents of cp genomes in Cedreloideae (37.77%-38.01%) are greater than those of the Melioideae (37.18%-37.70%) (**Table 2**). *K. senegalensis* exhibits the highest GC content in chloroplast genomes (38.01%), while *M. azedarach* has the lowest GC content.

**Table 2:** The sequence statistic of cp genomes of Meliaceae.

Species	CP genome	LSC	SSC	IR	GC content (%)			
	Length (bp)	Length (bp)	Length (bp)	Length (bp)	Total	LSC	SSC	IR
*K. senegalensis*	159,794	87,419	18,303	27,036	38.01	36.07	32.42	42.82
*K. grandifoliola*	159,794	87,419	18,303	27,036	37.95	36.07	32.42	42.82
*K. ivorensis*	159,750	87,358	18,320	27,036	37.94	36.08	32.46	42.82
*K. anthotheca*	159,841	87,444	18,327	27,035	37.93	36.07	32.44	42.81
*S. mahagoni*	159,276	86,958	18,214	27,052	37.95	36.10	32.46	42.78
*S. macrophylla*	159,276	86,958	18,214	27,052	37.95	36.1	32.46	42.78
*X. rumphii*	159,282	87,381	17,967	26,967	37.87	35.94	32.33	42.82
*X. moluccensis*	159,319	87,321	17,998	27,000	37.87	35.96	32.29	42.82
*X. granatum*	159,410	87,345	18,041	27,012	37.86	35.95	32.29	42.80
*Ca. guianensis*	159,483	87,051	18,278	27,077	37.91	36.01	32.42	42.80
*E. utile*	159,650	87,131	18,463	27,028	37.77	35.97	32.17	42.78
*E. cylindricum*	159,635	87,113	18,560	26,981	37.90	35.96	32.14	42.77
*E. angolense*	159,682	87,150	18,446	27,043	37.81	35.92	32.21	42.75
*E. candollei*	159,638	87,170	18,440	27,014	37.82	36.03	32.37	42.80
*Lo. swynnertonii*	158,881	86,506	18,249	27,063	38.00	36.17	32.42	42.86
*Lo. trichilioides*	159,381	86,989	18,454	26,969	37.98	36.14	32.51	42.76
*T. fargesii*	159,578	87,176	18,372	27,015	37.92	36.08	32.33	42.78
*T. ciliata*	159,617	87,100	18,399	27,059	37.89	36.05	32.26	42.76
*T. sinensis*	159,242	86,852	18,346	27,022	37.88	36.02	32.19	42.79
*Ce. odorata*	158,558	86,390	18,380	26,894	37.87	36.04	32.19	42.78
*L. laurentii*	160,470	87,685	18,681	27,052	37.66	35.72	31.98	42.77
*L. thompsonii*	160,499	87,707	18,676	27,058	37.66	35.71	31.97	42.77
*L. cedrata*	160,609	87,884	18,591	27,067	37.64	35.67	32.04	42.78
*Tu. africanus*	159,881	87,525	18,228	27,064	37.65	35.63	32.10	42.77
*La. domesticum*	159,573	87,062	18,515	26,998	37.69	35.77	31.89	42.78
*Ag. odorata*	160,978	88,146	18,654	27,089	37.52	35.53	31.85	42.73
*Ap. polystachya*	160,236	87,484	18,672	27,040	37.58	35.59	31.84	42.78
*Ci. cinerascens*	160,590	87,855	18,623	27,056	37.70	35.76	32.16	42.74
*H. velutina*	160,386	88,197	18,273	26,958	37.66	35.66	32.05	42.82
*M. azedarach*	160,393	87,598	18,709	27,043	37.18	35.37	31.38	42.68
*A. siamensis*	160,477	87,790	18,651	27,018	37.54	35.57	31.71	42.75
*A. indica*	160,876	88,139	18,629	27,054	37.49	35.53	31.64	42.72

A total of 135 genes were annotated in the cp genomes of Meliaceae species, comprising 90 protein-coding, 37 tRNA, and 8 rRNA genes (**Supplementary Table 1**). The genes were categorized into four groups: 76 self-replicating genes, 45 photosynthesis-related genes, 6 other genes, and 8 genes of unknown function.

### 3.2 Phylogenetic analyses

**Figure 2:**
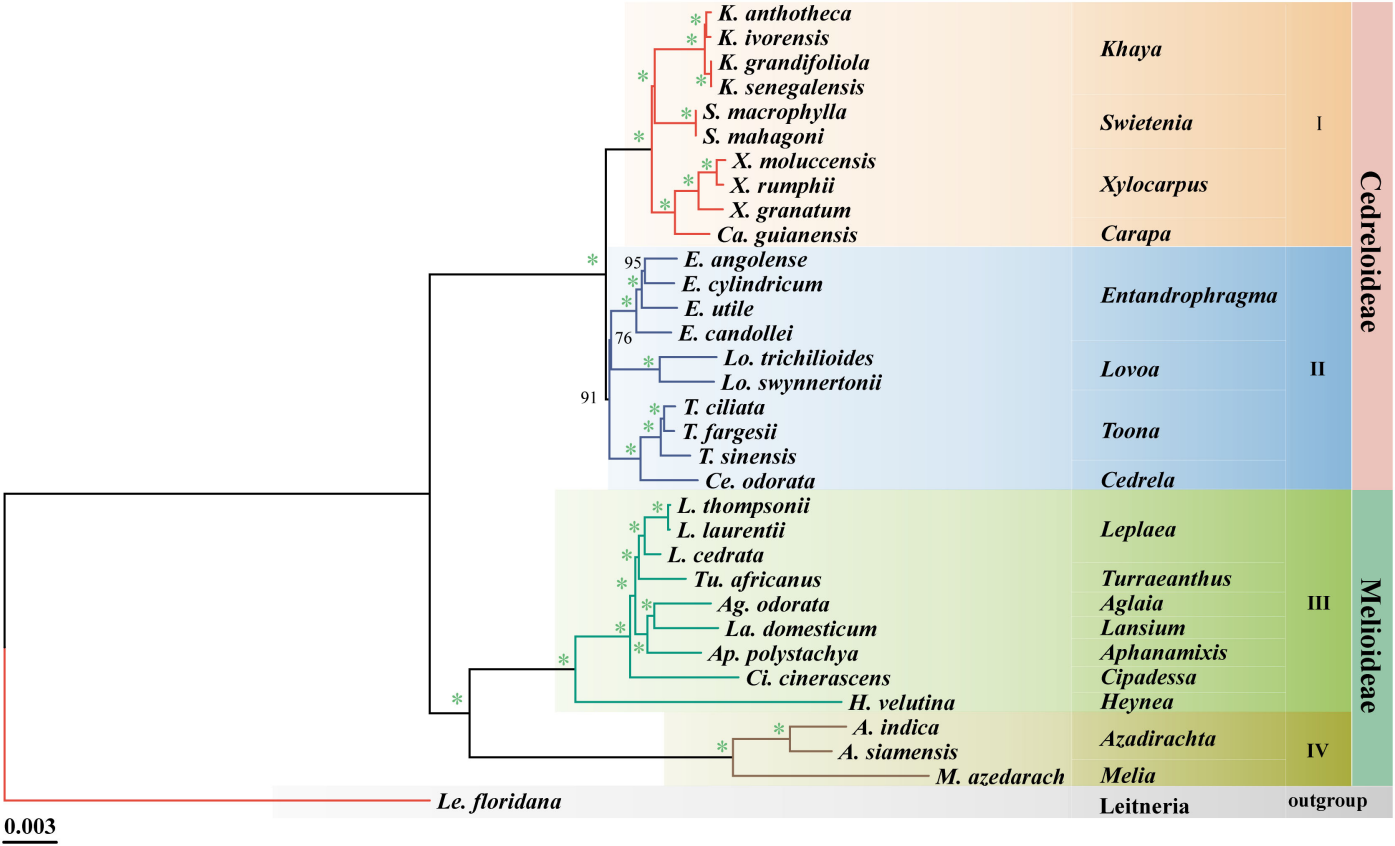
Phylogenetic tree of the cp genome of Meliaceae based on the maximum likelihood (ML) analysis. Bootstrap values (BS) are indicated at branch nodes, with an asterisk indicates support of 100% bootstrap.

The optimal model was obtained based on TVM + F + I + I + R5 using PhyloSuite, and the ML tree among 33 species of Meliaceae was reconstructed based on the cp genomes. In the phylogenetic tree, 90% of the branches had 100% support, and all branches had > 75% support (**Figure 2**). This indicates that the clustering results were highly reliable. Our results were consistent with previous reports (Muellner et al., 2016), which divided Meliaceae into two subfamilies, Cedreloideae (20 species across 8 genera) and Melioideae (12 species across 9 genera). Meliaceae can be further divided into four clades (I-IV). However, there is a slight difference compared to previous reports (Guo et al., 2018) in the location of *Swietenia*. In clade I, *Khaya* and *Swietenia* (BS = 100%), as well as *Xylocarpus* and *Carapa* (BS = 100%), each form two distinct monophyletic clades, respectively. Clades II-IV exhibited little controversy. In clade II, *Toona* clustered with *Cedrela* (BS = 100%), while *Entandrophragma* clustered with *Lovoa* (BS = 76%). Among them, *T. fargesii* is more closely related to *T. ciliata* than to *T. sinensis* within *Toona.* In clade III, *Leplaea* was found to share a close relationship with six other genera (*Turraeanthus*, *Lansium*, *Aglaia*, *Aphanamixis*, *Cipadessa*, and *Heynea*). Furthermore, *Melia* and *Azadirachta* formed the IV clade (**Figure 2**).

### 3.3 Divergence time estimation

**Figure 3:**
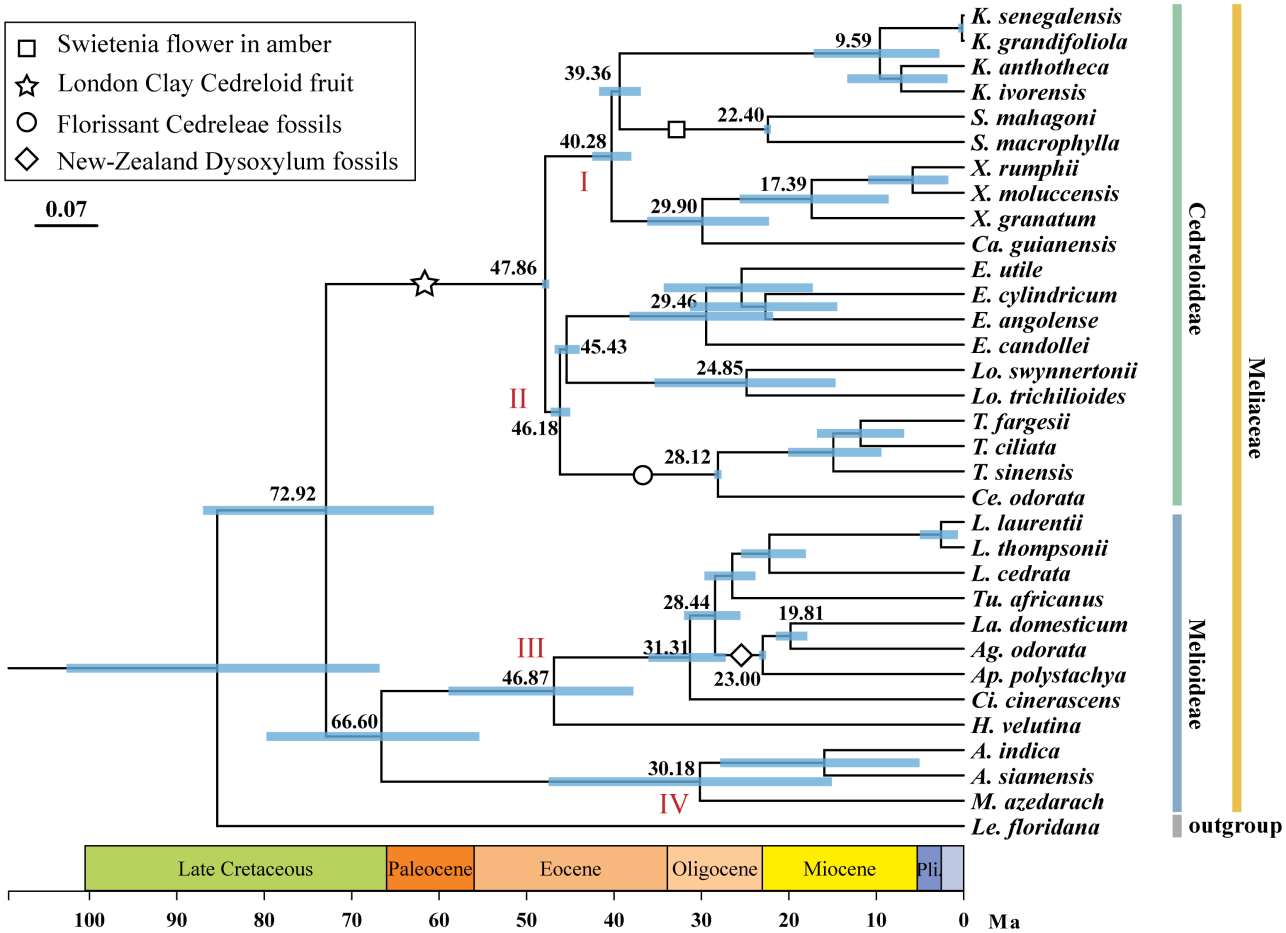
Divergence times of major clades within Meliaceae. The mean and 95% HPD intervals of the divergent time (Ma) are presented for select major nodes, indicated by blue lines. Black and white symbols represent the four calibration points.

The differentiation times of Meliaceae were shown in **Figure 3**. The ESS values, as tested by Tracer, were significantly higher than 200, indicating the credibility of the results obtained. The common ancestor of 32 Meliaceae species can be traced back to 72.92 million years ago (Ma) (95% HPD: 60.62-87.01). The Cedreloideae subfamily differentiated at the Eocene epoch, approximately 47.86 Ma (95% HPD: 47.82-48.22), later than the Melioideae subfamily (66.60 Ma, 95% HPD: 55.41-79.73 Ma). The four clades (I, II, III, and IV) differentiated at approximately 40.28 Ma, 46.18 Ma, 46.87 Ma, and 30.18 Ma, respectively. The oldest divergence of *Khaya* species, including *K. senegalensis*, *K. grandifoliola*, *K. ivorensis*, and *K. anthotheca*, from their common ancestor occurred at 9.59 Ma (95% HPD: 2.79-17.14 Ma). In contrast, *Heynea* was the earliest to diverge at 46.87 Ma (95% HPD: 37.78-58.92 Ma). Other Meliaceae species diverged from the Oligocene and the Miocene epochs. Specific node information was detailed in **Supplementary Table 2**.

### 3.4 Comparative genomic analysis

#### 3.4.1 IR contraction and expansion

**Figure 4:**
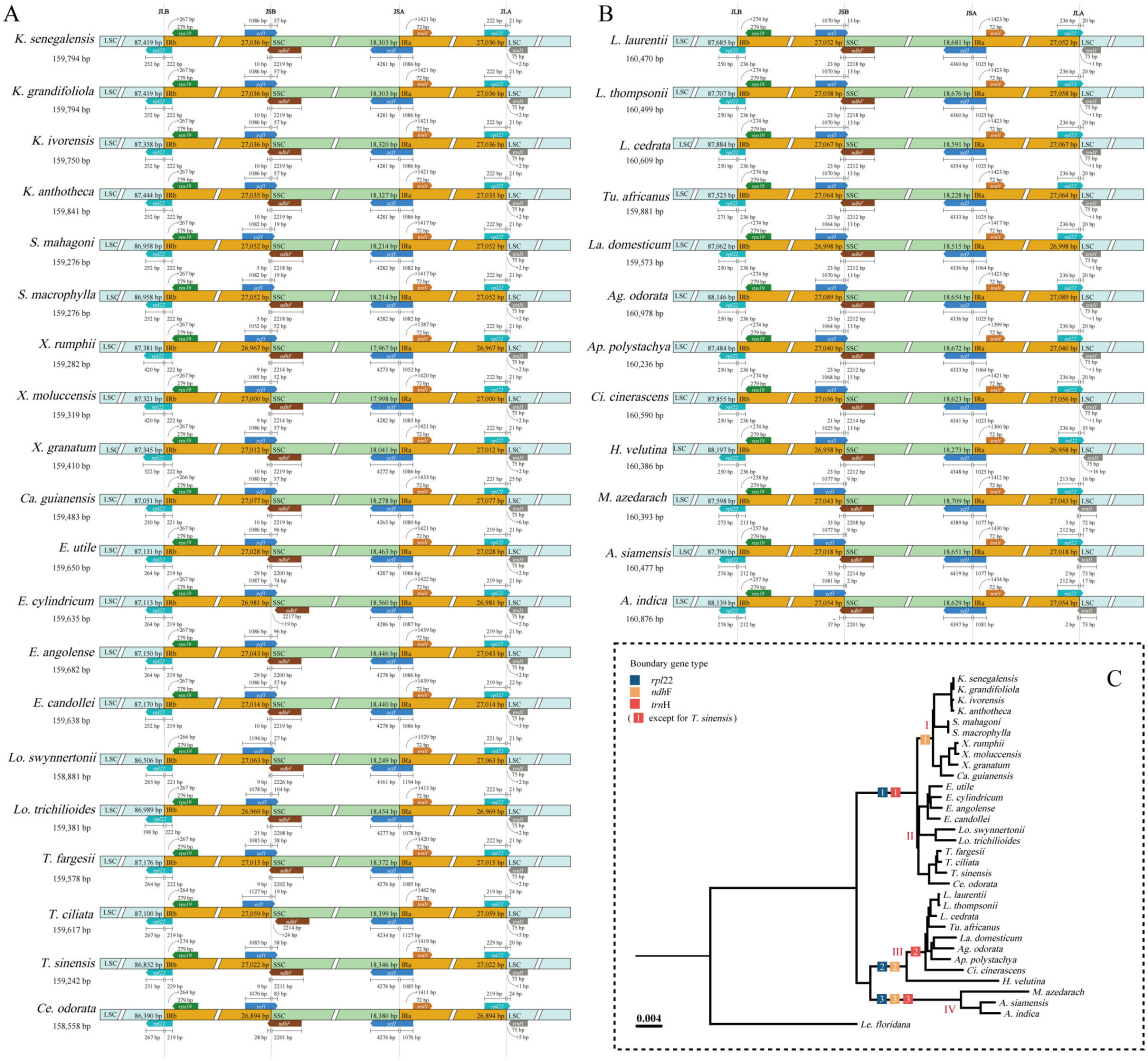
Changes in IR boundary analysis of 32 Meliaceae species. **(A)** Cedreloideae; **(B)** Melioideae; **(C)** Variations in gene locations near the IR boundary were depicted in in the Phylogeny tree.

During the evolution of the cp genome, the four boundaries (LSC, SSC, IRB, and IRA) have undergone expansions and contractions, facilitating the entry of specific genes into IR, LSC, and SSC. Boundary expansion and contraction analyses revealed slight variations at the boundary locations among Meliaceae species (**Figure 4**). In all Meliaceae cp genomes, the LSC/IRB (JLB) boundaries and the LSC/IRA (JLA) boundaries were located within the *rpl*22 gene. Notably, the length of *rpl*22 in the ‘*X. rumphii*-*Ca. guianensis*’ clade increased significantly. In clades I and II, *rpl*22 spanned the JLB boundary, with 219-229 bp located in IRB. Similarly, in clades III and IV, *rpl*22 also extended across the JLB boundary, with 236 bp and 212-213 bp, respectively, within the IRB. The SSC/IRA (JSA) boundaries were located within the *ycf*1 gene. The distances between the ends of *ycf*1 and the JSA boundaries ranged from 1023 to 1194 bp. Excluding *E. cylindricum* and *T. ciliata*, where the *ndh*F genes were entirely located within the SSC, the *ndh*F genes of other Meliaceae cp genomes were found in the SSC/IRB (JSB). Specifically, the *ndh*F in the I, III, and IV clades crossed the JLB boundary, with 9-10 bp, 21-23 bp, and 33-37 bp located in the IRB, respectively. In the I and II clades, with the exception of *T. sinensis*, where *trn*H was situated 1 bp away from the JLA, *trn*H was found in the LSC, positioned 2-6 bp away from the JLA. In the III clade, excluding *Heynea velutina*, where *trn*H was located 16 bp away from the JLA, *trn*H was situated just 1 bp to the right of the JLA boundary. In clade IV, *trn*H expanded into the IRA by 2-3 bp.

#### 3.4.2 Comparison of diversity in cp genomes

**Figure 5:**
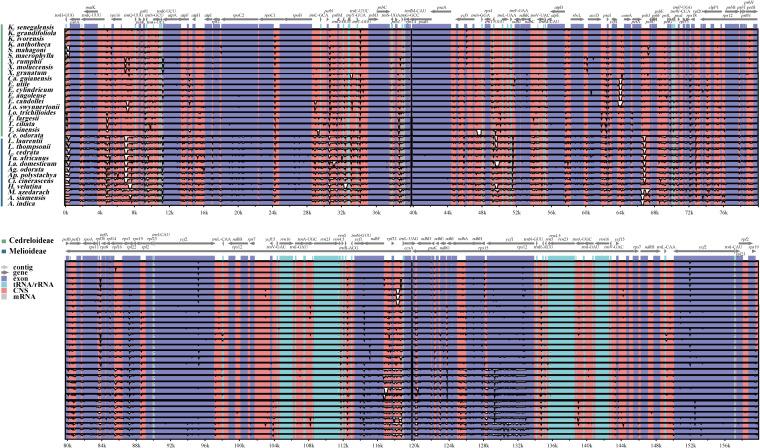
Visualization of the alignment of cp genome sequences Meliaceae species. The vertical scale represents the percentage of identity, ranging from 50% to 100%.

In this research, the cp genome of *K. senegalensis* served as the reference. The cp genomes of 32 Meliaceae species were compared using mVISTA. The results (**Figure 5**) indicate that the IR showed higher conservation compared to both the LSC and SSC. Moreover, the coding regions demonstrated greater conservation than the non-coding regions (CNS). High levels of diversity were observed in CNS, particularly in the *trn*H*-psb*A*, trn*K*-rps*16, *rps*16*-trn*Q*, pet*N*-psb*M*, psb*Z*-trn*G, and *ndh*F*-rpl*32*, rpl*32*-trn*L regions. Additionally, several genes, including *mat*K*, rpo*C2*, ndh*F, and *ycf*1, exhibited variable regions.

**Figure 6:**
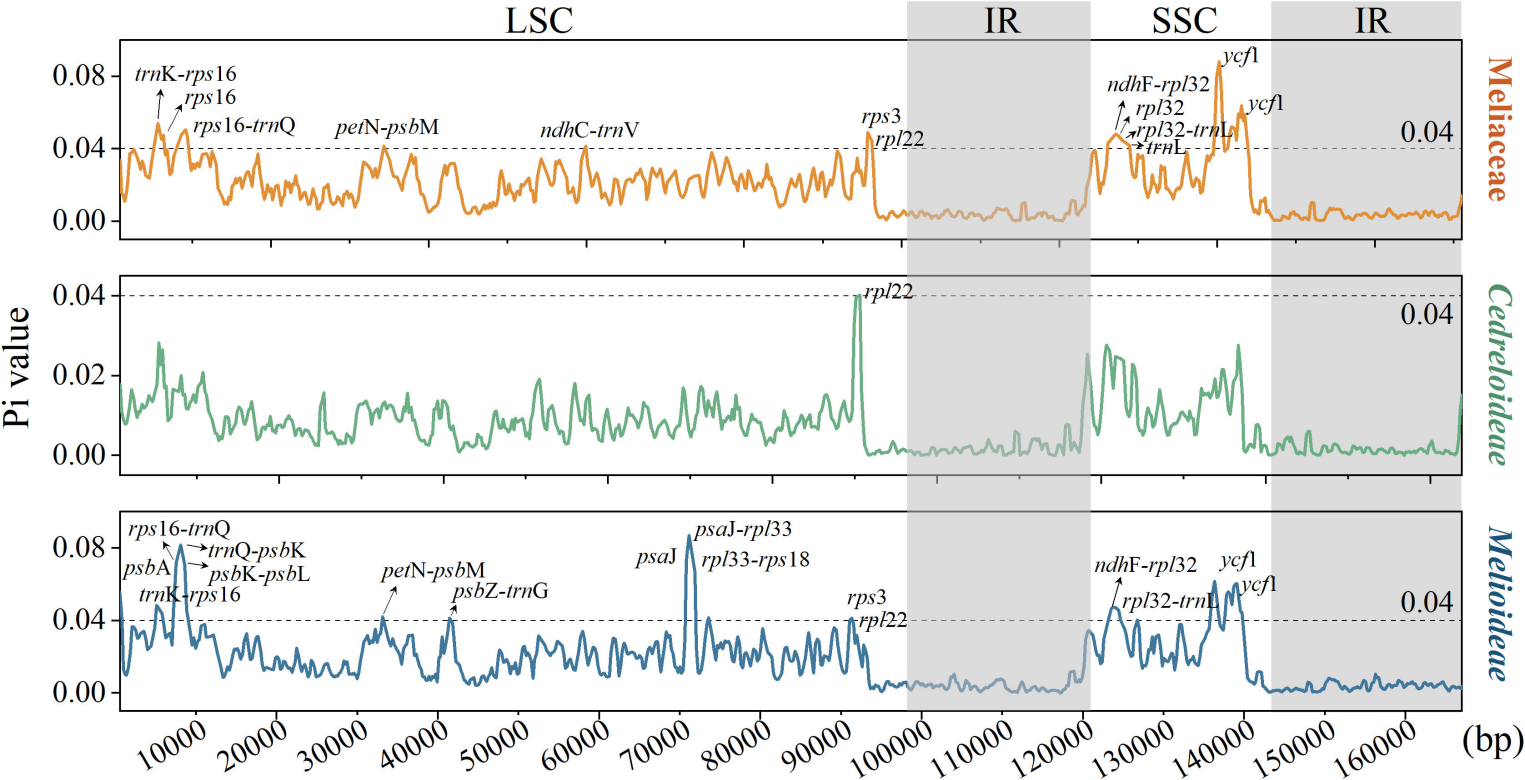
Nucleotide polymorphism analysis of cp genome within Meliaceae, Cedreloideae, and Melioideae.

To assess sequence variation within the cp genomes of Meliaceae, we employed DNAsp software to calculate nucleotide diversity of the 32 cp genomes. The results of the sliding window analysis (**Figure 6**) indicated that Pi ranged from 0 to 0.0881 (for *ycf*1), with a mean value of 0.01641. A total of 13,062 polymorphic sites were detected. Using Pi > 0.04 as the threshold, twelve regions with HVRs, including *ycf*1, *trn*K*-rps*16*, rps*16*-trn*Q*, ndh*F*-rpl*32*, rps*16*, rpl*32*, rpl*32*-trn*L*, rpl*22*, trn*L*, pet*N*-psb*M, and *ndh*C*-trn*V, were identified in Meliaceae. Among the variable sites, Melioideae has 15 sites with Pi > 0.04, whereas Cedreloideae has only one such site (*rpl*22). All identified HVRs were located in LSC and SSC. Furthermore, we analyzed the variation within certain genera of Meliaceae. The results showed (**Supplementary Figure 1**) that the individual genera in Meliaceae exhibited little variation, with pi values being less than 0.04, except for the *ycf*4-*cem*A of *Lovoa* (Pi = 0.05) and the *trn*K-*rps*16 of *Azadirachta* (Pi = 0.055). These loci can be further utilized for the study of gene polymorphisms.

#### 3.4.3 Simple sequence repeats

**Figure 7:**
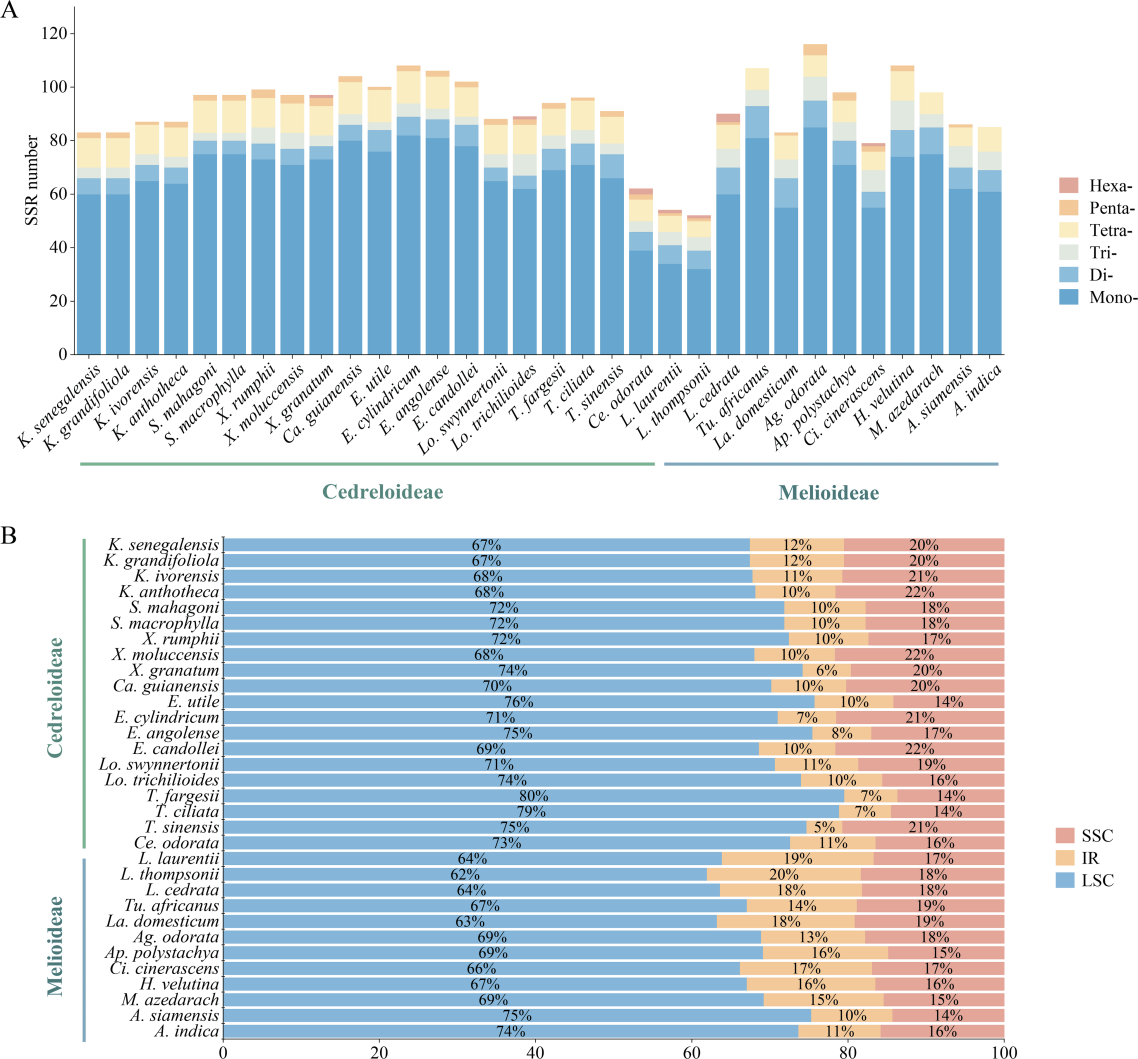
SSR type in the cp genomes of Meliaceae. **(A)** Number of SSRs in different species. **(B)** Percentage distribution of SSRs in different genomic regions.

Using MISA software for simple sequence repeat analysis, the findings revealed that the number of SSRs in Meliaceae ranged from 52 (*Leplaea thompsonii*) to 116 (*Ag. odorata*) (**Figure 7A**). There are six types of nucleotides in Meliaceae, comprising mononucleotide repeats through hexanucleotide repeats. Among these, mononucleotide repeats were the most abundant, ranging from 32 to 85, with A/T bases being the predominant bases. In Cedreloideae, tetranucleotide repeats accounted for the second-highest percentage. In contrast, within Melioideae, nine species exhibited a greater number of dinucleotide repeats than tetranucleotide repeats. Additionally, the AC/GT bases were found exclusively in the III clade; only *Toona* and *Cedrela* each had 2 AG/CT bases, while other species contained 0-1 AG/CT bases. Tetranucleotide repeats were the most abundant, comprising nine types; however, the AAGT/ACTT bases were limited to the ‘*K. senegalensis*-*Lo. trichilioides*’ clade, and only *Entandrophragma candollei* exhibited the AATT/AATT base. We identified five kinds of pentanucleotides within Meliaceae, of which AAAAG/CTTTTT occurred only in *Xylocarpus* and *Carapa*, while AAAAT/ATTTT was found exclusively in *Ag. odorata*, *Azadirachta siamensis*, and *Aphanamixis polystachya*, and each species of Cedreloideae contained AAATT/AATTT bases. Furthermore, only *Ag. odorata* possessed AATTC/AATTG bases. Hexanucleotides were detected in a limited number of Meliaceae species (**Supplementary Table 3**).

Most SSRs were located in the LSC (61.97%-79.55%), while a smaller percentage were distributed in the SSC (4.6%-19.72%) and IR (13.61%-21.65%) (**Figure 7B**). The proportion of SSRs in Cedreloideae species in the SSC was higher than that in the IR. However, some Melioideae species exhibited an SSR percentage in the IR that was greater than or equal to that in the SSC. The SSR proportions were consistent within the same genus, with one exception noted in *Leplaea*.

#### 3.4.4 Codon preference

**Figure 8:**
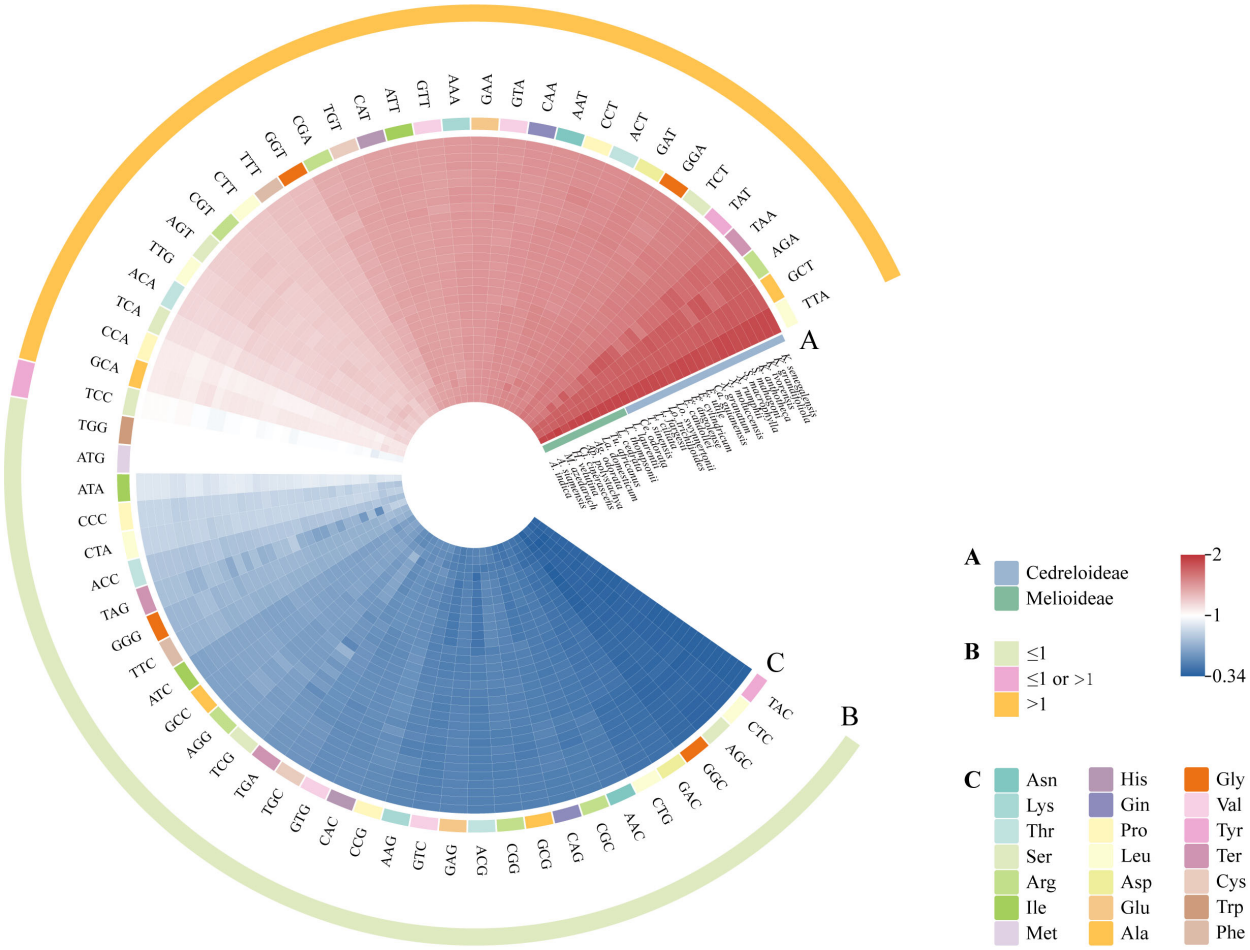
Codon usage mode of cp genomes in Meliaceae. The color intensity in the heatmaps represented the magnitude of the RSCU values. **(A)** Species from different clades are represented in different colors. **(B)** Yellow circles represent codons with RSCU > 1, pink circles represent codons with RSCU > 1 or RSCU ≤ 1, and green circles represent codons with RSCU ≤ 1. **(C)** Different colored rectangles indicate different types of amino acids encoded.

The results of RSCU values revealed that the same codons from all Meliaceae species had similar color distributions and codon usage patterns (**Figure 8**; **Supplementary Table 4**). These codons encode for 20 amino acids (excluding stop codons). Except for methionine and tryptophan, which are encoded by single codons each, all other amino acids are encoded by multiple codons. The most frequently used codons are UUA, GCU, and AGA. Meliaceae cp genomes contain 30-31 codons with RSCU > 1. Among the 32 Meliaceae species, seven species (all of which belong to Cedreloideae) contained 31 codons with RSCU > 1. The codons with RSCU > 1 corresponded to amino acids that are preferentially coded, such as AUG, UUA, and GCU. Except for the codons UUG, AUG, and UCC, the third base of the remaining high-frequency codons terminates with either an A or U base.

## 4 Discussion

### 4.1 Cp genome structure and gene of Meliaceae

In this research, we sequenced the cp genomes of three *Toona* and performed comparative analyses with 29 other Meliaceae cp genomes from NCBI. Among them, the cp genome of *T. fargesii* is reported for the first time. The cp genomes of *Toona* exhibit typical tetrameric structures, comprising LSC, SSC, IRA, and IRB. The cp genomes of *Toona* ranged from 159,242 bp to 159,617 bp in length, with a total GC content varying from 37.88% to 37.92%. Within Meliaceae, the lengths of the cp genome, LSC, SSC, and IR were 158,558 bp-160,978 bp, 86,390 bp-88,197 bp, 17,967 bp-18,709 bp, and 26,894 bp-27,089 bp, respectively. The total GC content ranged from 37.18% to 38.01%. Furthermore, the GC content in the IR (42.68%-42.86%) was significantly higher than that in the LSC (35.37%-36.17%) and the SSC (31.38%-32.51%). This finding is consistent with previous reports in other plant species (Wang et al., 2022). This difference may be attributed to the presence of rRNA genes with higher GC content in the IR (Li and Zheng, 2018), while the lower GC content in the SSC may be associated with the presence of the *ndh*H gene located within this region. Additionally, species within the Cedreloideae subfamily exhibited a higher GC content (37.77%-38.01%) compared to those within the Melioideae subfamily (37.18%-37.70%). Hao et al. (2022) found that the evolutionary rate has a negative correlation with GC content. We speculate that Cedreloideae evolved more recently than Melioideae, which is consistent with the estimation of divergence time in this study. All cp genomes of Meliaceae were annotated to 135 genes, primarily involved in photosynthesis, transcription, and translation, which is consistent with general angiosperms. These results indicate that the genome structure and gene numbers are relatively conserved among Meliaceae plants.

### 4.2 Phylogenetic analyses

Considerable research has been conducted on the origin and evolution of Meliaceae (Wikström et al., 2001; Muellner et al., 2007; Bell et al., 2010; Koenen et al., 2015). However, nearly all studies have constructed phylogenetic trees using plastid fragments and nuclear internal transcribed spacers. While these results identified the major clades within Meliaceae, support for certain clades composed of similar species was relatively low (Muellner et al., 2003, 2007; Muellner and Mabberley, 2008; Muellner et al., 2016; Guo et al., 2018). The use of complete cp genomes for classification has been widely recognized across several plant taxonomic groups, such as *Panax* (Xia et al., 2025) and *Cyclorhiza* (Song et al., 2025). Consequently, we constructed a phylogenetic tree of 33 complete cp genomes, including 32 distinct Meliaceae species, with *Le. floridana* serving as the outgroup. Phylogenetic analyses strongly support the classification of these 32 Meliaceae species into the Cedreloideae and Melioideae subfamilies. Morphological evidence further supports this division: each locule of Melioideae plants contains one or two ovules, while each ovule of Cedreloideae plants contains three or more ovules (Laino Gama et al., 2021). Furthermore, the relationships identified in our results generally agree with those reported in previous studies (Muellner et al., 2016), with most clades receiving greater support; however, subtle differences in the positioning of *Swietenia* were also observed. Previous studies have indicated that *Swietenia*, along with *Khaya, Xylocarpus*, and *Carapa*, formed the tribe Xylocarpeae (Laino Gama et al., 2021). These four genera share several morphological traits, such as a corolla with contorted aestivation and anthers inserted on the inner side of the staminal tube (Laino Gama et al., 2021). Based on five cpDNA loci, earlier taxonomy by Guo et al. (2018) suggested that *Swietenia* was more closely related to the *Xylocarpus* and *Carapa* clade than to *Khaya*. In contrast, our analysis of complete cp genomes indicates that *Khaya* and *Swietenia* formed a sister group, while *Xylocarpus* and *Carapa* constituted another sister group in our analysis. This difference may be due to the lack of sufficient information for genes or gene spacers when compared to complete cp genomes. All genera within Meliaceae exhibited strong monophyly, consistent with the findings of Oyedeji Amusa et al. (2024). However, some genera within Melioideae were represented by only one species in our analysis. We recommend collecting additional species to enhance the understanding of phylogenetic relationships within Meliaceae.

### 4.3 Divergence time of Meliaceae

In this study, the earliest divergence in the Meliaceae to have approximately occurred in 72.92 Ma (95% HPD: 60.62-87.01 Ma). Previous studies have reported earlier divergence time (67.5 Ma, 95% HPD: 51.8-86.4 Ma) (Guo et al., 2018), later divergence times (79.60 Ma; 80.4 Ma, 95% HPD: 69.66-89.77 Ma; 95% HPD:69.1-91.6 Ma) (Muellner et al., 2016; Koenen et al., 2015), and the divergence time that are consistent with our findings (73.0 Ma, 95% HPD: 61.4-83.9 Ma) (Muellner et al., 2007) (**Supplementary Table 5**). These divergence times did not differ much from our results, suggesting the reliability of our result. Most current species of Meliaceae were distributed in tropical or subtropical regions, inhabiting diverse habitats ranging from rainforests and mangrove swamps to semi-deserts (Yadav et al., 2015). Muellner et al. (2006) indicated that Meliaceae likely originated from Gondwanan and experienced an “out-of-Africa” dispersal through different routes. In the late Cretaceous, the separation of Africa and South America began at about 135-130 Ma with seafloor spreading in the South Atlantic and ended at 90-85 Ma (Jones, 1987). Subsequently, the frequency of plant dispersal gradually decreased until the Miocene epoch (Morley, 2003). Therefore, we infer that the breakup of Gondwana and subsequent continental drift led to the initial diversification of Meliaceae (about 72.92 Ma, 95% HPD: 60.62-87.01 Ma). Koenen et al. (2015) showed that Meliaceae experienced a rapid differentiation event. This rapid radiation has resulted in significant morphological overdiversification and taxonomic confounding within this family. Our results are similar to those of Koenen et al. (2015), indicating that Meliaceae species rapidly diverged from the Late Oligocene or the Early Miocene epochs. Recent speciation events may be related to the substantial fluctuations in sea level and CO_2_ concentrations (Carrapa et al., 2019). Plate subduction along the Pacific margin and the collision of new plates resulted in the formation of the Andes between 34 and 65 Ma (Hoorn et al., 2010). The construction of the mountains peaked for the first time during the late Oligocene to early Miocene (approximately 23 Ma). As the mountains uplifted, rainfall increased on the eastern side, promoting changes in the ecological environment (Hoorn et al., 2010). This transformation may have converted some rainforest habitats into coastal wetlands, thereby providing opportunities for Meliaceae species to colonize intertidal environments, which likely contributed to increased speciation rates within this family.

### 4.4 Comparative genomic analysis

Contraction and expansion of IRs are occurrences in plant evolution, affecting the length and copy number of cp genomes, leading to gene deletions, duplications, and the generation of pseudogenes. *Ycf*1 and *rps*19 have been observed to originate from the contraction and expansion of IR in angiosperms (Zhu et al., 2016). Consistent with observations in most angiosperms, *ycf*1 in Meliaceae begins in IR and then extends into SSC, resulting in the formation of pseudogenes. *Rps*19 is typically positioned at the JLA and JSB boundaries. The cp genomes of Meliaceae have undergone an expansion of IR, letting *rps*19 entirely into the IR. This pattern is comparable to observations in certain species from the Hydrangeaceae family (Raman et al., 2023). Contraction and expansion analyses of the IR boundaries of the cp genome in Meliaceae exhibited a high level of conservation, with the exception of the JLB junction in *E. cylindricum* and *T. ciliata*, and the JLA junction in the IV clade (*Melia* and *Azadirachta*). *Khaya* and *Swietenia* showed minimal shifts in these boundaries. This finding indicates that these two genera are the most conserved within Meliaceae.

Comparative analysis of the Meliaceae cp genomic sequences revealed the presence of several HVRs. These HVRs were identified in the interstitial regions of *trn*H*-psb*A*, trn*K*-rps*16, *rps*16*-trn*Q*, pet*N*-psb*M*, psb*Z*-trn*G, *ndh*F*-rpl*32, and *rpl*32*-trn*L, as well as within the gene regions such as *mat*K*, rpo*C2*, ndh*F, and *ycf*1. The majority of these HVRs were primarily concentrated in LSC and SSC. The HVRs in CNS were more abundant than those found in coding regions, similar to the patterns observed in other angiosperms (Shi et al., 2023). Among these, *rpl*32*-trn*L exhibits significant nucleotide mutations, making it an ideal molecular marker for low-order metaclassification studies in plants. *Trn*H*-psb*A and *mat*K are commonly employed in plant DNA barcoding studies; however, previous research has revealed that *trn*H*-psb*A (Fukuda et al., 2003) and *mat*K (Muellner et al., 2003) exhibit ordinary levels of universality and resolvability for species identification in Meliaceae.

To further elucidate the variations of HVRs, we analyzed the Pi values for Meliaceae, Cedreloideae, and Melioideae using a standardized threshold (Pi > 0.04). We identified twelve regions in Meliaceae cp genomes exhibiting high Pi values, including *ycf*1, *trn*K-*rps*16, *rps*16-*trn*Q, *ndh*F-*rpl*32, *rps*16, *rpl*32, *rpl*32-*trn*L, *rpl*22, *trn*L, *pet*N-*psb*M, and *ndh*C-*trn*V. Furthermore, the findings indicate that the distribution of polymorphic sites changes significantly among different subfamilies. Among these, the sites of *ycf*1, *trn*K-*rps*16 and *ndh*F-*rpl*32 showed the highest Pi values. In Melioideae, fifteen regions were identified with high Pi values. However, only one region, *rpl*22, was identified as having high Pi values in Cedreloideae. This observation may be attributed to differing rates of evolution among the various subfamilies. Moreover, *trn*K-*rps*16 also exhibited higher variability detected in the *Azadirachta* genus. The Pi values among species within all genera were low, suggesting that the degree of species differentiation within genera is low. Most of these HVRs were located within LSC and SSC, with intergenic regions exhibiting greater variability compared to gene regions, potentially due to reduced selective pressures. Plastid barcoding markers have been employed in Meliaceae (Erickson et al., 2008), confirming their stability for species identification. For instance, Koenen (2011) revealed that *ycf*1 has been effectively utilized as a molecular markers, providing more informative data than conventional markers in phylogenetic analyses of Meliaceae.

Simple repetitive sequences are DNA fragments characterized by multiple repetitions of one to six nucleotides. These SSRs can be utilized in plant population genetics and evolutionary studies due to their distinct parental genetic characteristics (Su et al., 2023). We identified between 52 and 116 SSRs within Meliaceae, with mononucleotide repeats comprising the majority, consistent with earlier research (Sun et al., 2024). The predominant presence of A/T in these SSRs can be attributed to polyadenylation at the 3’ end of mRNA following transcription. Alternatively, this may result from the relative ease with which the A/T-rich strand separates from its complementary G/C-rich strand during DNA replication, which subsequently increases the likelihood of strand slippage and mismatch (Dodsworth et al., 2014). All examined cp genomes possessed mononucleotides to pentanucleotides repeats, with a minority also containing hexanucleotides repeats. Notably, the AAAAG/CTTTT repeat was identified exclusively within the ‘*X. rumphii*-*Ca. guianensis*’ clade. Combined with the IR boundary analysis results, we suspect that this is caused by the length variation of *rpl*22 in this clade. Specific SSR types were only observed in certain species, such as AAC/GTT (*H. velutina*), AATTC/AATTG (*Ag. odorata*), AAAAGT/ACTTTT (*Ce. odorata*), AACGAT/ATCGTT (*Lo. trichilioides*), AAGAAT/ATTCTT (*L. cedrata*), and AATATT/AATATT (*Cipadessa cinerascens*). Furthermore, species belonging to some genera (*Leplaea* and *Aglaia*) were found to lack the ACAT/ATGT repeat. However, this analysis was conducted based on the 32 sequences we employed. Therefore, broader sampling is essential in order to fully enhance the accuracy and reliability of the results. The development of such a method will greatly contribute to SSR assessments. In addition, the pattern of SSRs varied little within *Leplaea*, especially *L. thompsonii* and *L. cedrata*, which is similar to their phylogenetic results. Two distinct patterns of SSR distribution were evident in Meliaceae. The first pattern indicated that SSRs are predominantly located in LSC, followed by SSC, with IR exhibiting the least SSR concentration. In contrast, a second pattern revealed that while LSC remained the primary region for SSR distribution, the presence of SSRs in the IR was comparable to that in the SSC. With the exception of seven Melioideae species, the majority of species in Meliaceae conformed to the first distribution pattern. The concentration of SSRs in LSC may correlate with increased gene density and functional diversity, whereas the limited distribution of SSRs in IR could be linked to its role in maintaining genome stability. Variations in repeat types indicate diverse evolutionary histories, likely influenced by shifting selective pressures and environmental factors (Martin and Orgogozo, 2013). These SSRs represent promising candidate molecular markers for species within Meliaceae.

Codon preference is prevalent in plants and is influenced by various factors, including gene expression levels, GC content, amino acid conservation, and transcriptional selection. These factors play an important role in species evolution and genetic studies (Quax et al., 2015). RSCU is the ratio of the actual frequency of a codon to the theoretical frequency. An RSCU value greater than 1 indicates that the codon is utilized more frequently than other synonymous codons, indicating it as a high-frequency codon. Conversely, an RSCU value less than 1 suggests that the codon is a low-frequency codon. An RSCU value equal to 1 means there is no codon usage bias (De Oliveira et al., 2021). In this study, we observed that the highest frequency codons predominantly end with A/U. Our findings align with previous studies on *Veratrum* (Zhang et al., 2022) and *Aconitum* (Wang et al., 2024). The preference for A/T-ending codons may correlate with mRNA stability and translational efficiency, as these codons potentially reduce the energy required for mRNA folding, thereby accelerating translation. Specifically, the TCC codon in certain species exhibited an RSCU > 1, all of which belong to Cedreloideae. This phenomenon may result from differing mutation pressures and natural selection across clades, leading to distinct codon usage biases (Hao et al., 2022).

## 5 Conclusions

In this study, we sequenced and assembled the complete cp genomes of *Toona*. These cp genomes, along with 29 previously reported cp genomes, were comparatively analyzed within Meliaceae. Based on 32 Meliaceae complete cp genomes, we constructed an ML phylogenetic tree that exhibited high bootstrap support. The results showed that the cp genomes of 32 Meliaceae species ranged in size from 158,558 bp to 160,978 bp. These cp genomes were annotated to contain 135 unique genes. Divergence time estimation revealed that most Meliaceae species differentiated into Cedreloideae and Melioideae subfamilies (72.92 Ma, 95% HPD: 60.62-87.01 Ma) in the late Cretaceous, with many species showing recent differentiation. The pantropical distribution of Meliaceae may be attributed to long-distance dispersal, and speciation events may be explained by geological activities, fluctuations in global temperatures, sea level, and CO_2_ concentrations. The cp genomes of Meliaceae exhibited slight differences in genome size, IR boundaries, genomic structure, HVRs, and codon patterns. These differences were consistent with phylogenetic relationships, likely attributable to distinct environmental selection pressures and evolutionary histories. Additionally, comparative analysis of the cp genomes revealed that we identified 52-116 simple repeats (SSRs), some of which appeared only in specific groups; twelve highly variable regions were detected with Pi > 0.04. *Ycf*1, *trn*K-*rps*16, and *ndh*F-*rpl*32 showed the highest nucleotide polymorphisms in the cp genomes of Meliaceae, while *rpl*22 demonstrated high nucleotide polymorphisms in both subfamilies. These regions were utilized as candidate molecular markers for phylogenetic studies and species identification. Overall, these results can not only provide insights into the evolution and species identification of Meliaceae, but also establish a scientific foundation for the future systematic research within this family.

## Data Availability

The datasets presented in this study can be found in online repositories. The names of the repository/repositories and accession number(s) can be found in the article/**Supplementary Material**.
