# Supplementary figures and images for: Comparative analysis of chloroplast genomes on Meliaceae species: insights into the evolution and species identification
Source: Front Plant Sci. 2025 Mar 11;16:1536313. doi: 10.3389/fpls.2025.1536313 (PMC11933007; doi:10.3389/fpls.2025.1536313)

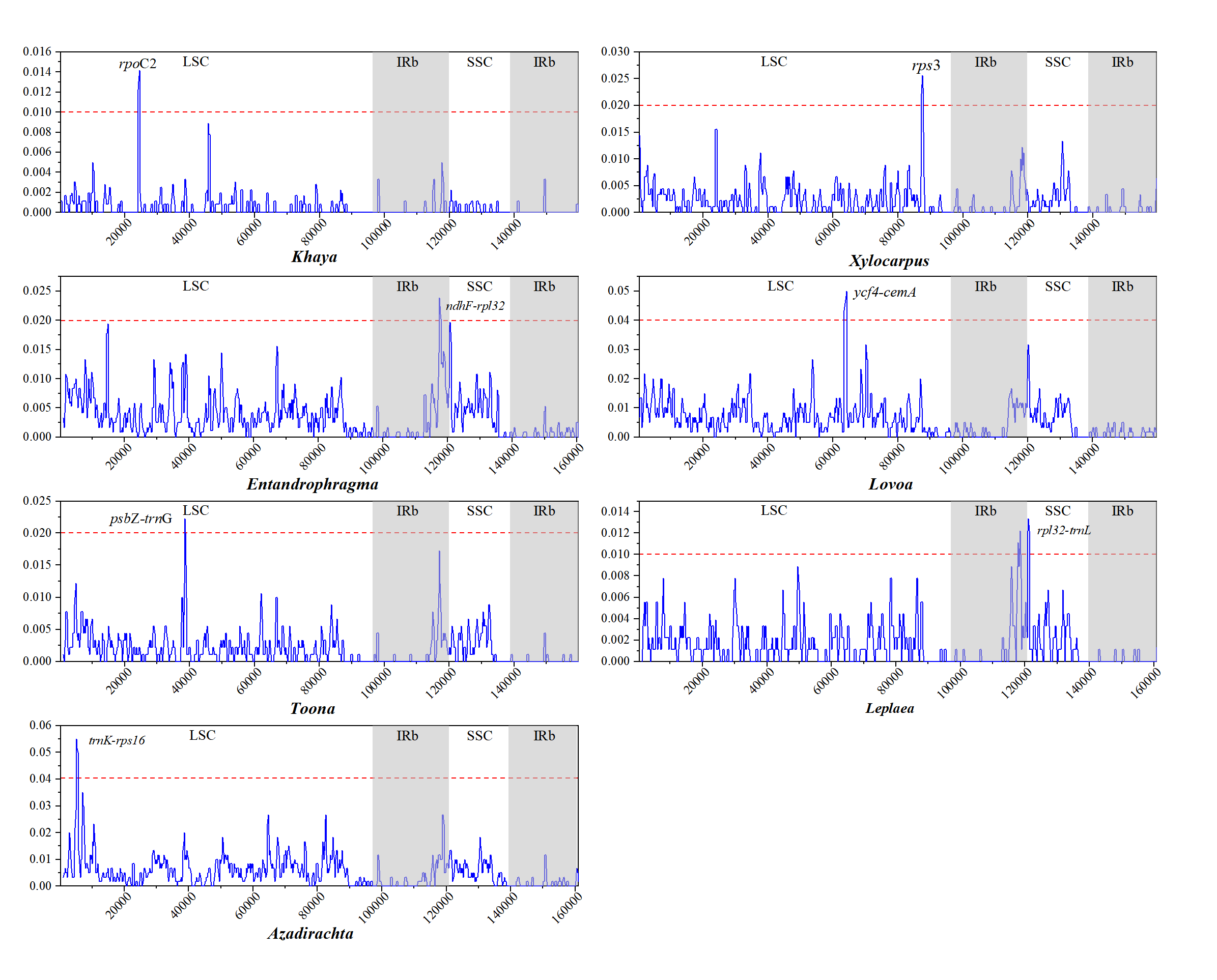

Supplement: Supplementary file 1 [file Image1.tif]
